# ESBL- and pAmpC-Producing *Salmonella* spp. and *Escherichia coli* O157:H7 Isolated from Bovine Carcasses in Türkiye

**DOI:** 10.3390/antibiotics15070658

**Published:** 2026-07-03

**Authors:** Pelin Koçak Kızanlık, Cemil Şahiner, Hafize Tuğba Yüksel Dolgun, Şükrü Kırkan, Filiz KöK, Ergün Ömer Göksoy

**Affiliations:** 1 Department of Food Hygiene and Technology, Faculty of Veterinary Medicine, Aydın Adnan Menderes University, 09010 Aydın, Türkiye; cemil.sahiner@adu.edu.tr (C.Ş.); fkok@adu.edu.tr (F.K.); eogoksoy@adu.edu.tr (E.Ö.G.); 2 Department of Microbiology, Faculty of Veterinary Medicine, Aydın Adnan Menderes University, 09010 Aydın, Türkiye; tugba.yuksel@adu.edu.tr (H.T.Y.D.); skirkan@adu.edu.tr (Ş.K.)

**Keywords:** antimicrobial resistance, β-lactamase genes, carcass contamination, foodborne pathogens, multidrug resistance, slaughterhouse

## Abstract

**Objectives**: Increasing antimicrobial resistance among foodborne pathogens, particularly extended-spectrum β-lactamase (ESBL) and plasmid-mediated AmpC β-lactamase (pAmpC) production, has become a major public health concern worldwide. This study aimed to determine the presence of *Salmonella* Enteritidis, *Salmonella* Typhimurium, and *Escherichia coli* O157:H7 in bovine carcasses and to evaluate their antibiotic resistance profiles together with ESBL and pAmpC resistance characteristics. **Methods**: A total of 300 bovine carcasses were examined for the presence of *Salmonella* spp. and *E. coli* O157:H7 using culture-based isolation methods following ISO 6579-1 and FDA guidelines, respectively. The isolates were confirmed by molecular methods, and *stx1*, *stx2*, *eae*, and *hly* were investigated in *E. coli* O157:H7 isolates. Antimicrobial susceptibility testing was performed according to EUCAST guidelines. ESBL and pAmpC production were determined phenotypically and subsequently characterized by molecular methods. **Results**: A total of 25 *Salmonella* spp. (32% *S*. Enteritidis and 68% *S*. Typhimurium) and 20 *E. coli* O157:H7 isolates were recovered from different bovine carcasses. *stx2* was the most frequently detected virulence gene. Of the 31 phenotypically ESBL-positive isolates, 29 carried at least one ESBL-associated gene. The predominant ESBL gene was *bla_CTX-M_* (79.3%), followed by *bla_TEM_* and *bla_SHV_* (37.9%). Among CTX-M gene groups, CTX-M-25 was the most prevalent (94.4%). Phenotypic pAmpC production was detected in 13 isolates, while 17 isolates carried at least one pAmpC-associated gene, with FOX identified as the predominant gene group. All isolates were resistant to pefloxacin, followed by gentamicin (93.3%) and cefoxitin (55.5%). Multidrug resistance was detected in 34 (75.6%) isolates. **Conclusions**: The detection of ESBL-producing, pAmpC-positive, and multidrug-resistant *Salmonella* spp. and *Escherichia coli* O157:H7 isolates in bovine carcasses indicates the presence of antimicrobial-resistant foodborne pathogens in the beef production chain. These findings highlight the need for continued monitoring of antimicrobial resistance and effective control measures at the slaughterhouse level.

## 1. Introduction

Foodborne diseases remain a major global public health concern. World Health Organization (WHO) reports that approximately 600 million people worldwide suffer from foodborne illnesses annually after consuming contaminated food, causing a global annual burden of 33 million disability-adjusted life years (DALYs) and 420,000 premature deaths [1]. Zoonotic pathogens most commonly responsible for these foodborne illnesses, particularly *Salmonella* spp. and Shiga toxin-producing *Escherichia coli* (STEC), have been reported among the five most common foodborne pathogens in the European Union (EU). According to the European Food Safety Authority (EFSA) and the European Centre for Disease Prevention and Control (ECDC) report, *S*. Enteritidis and *S*. Typhimurium are the most prevalent *Salmonella* serovars and approximately 40% of STEC infections are caused by *E. coli* O157 [2]. Bovine meat may serve as a vehicle for *Salmonella* transmission through contamination of carcasses by fecal material, internal organs and lymph nodes from infected animals [3].

Cattle are considered as the main reservoir of *E. coli* O157:H7, and carcass contamination generally occurs through fecal content during the slaughtering process. Furthermore, inadequate sanitation procedures and cross-contamination via food-contact surfaces and personnel facilitate the dissemination of this pathogen. These conditions make bovine meat an important source of salmonellosis and STEC infections [3,4,5]. Besides producing Shiga toxins (Stx1 and Stx2), *E. coli* O157:H7 possesses additional virulence genes associated with bacterial adhesion, colonization, and disease progression, including *eae* and *hly* [6]. Consequently, infections caused by virulent strains may result in severe clinical manifestations such as hemorrhagic colitis, hemolytic uremic syndrome (HUS), and thrombotic thrombocytopenic purpura (TTP) [7]. EFSA has identified *stx1*, *stx2*, and the adhesion-associated *eae* gene as key molecular markers for the characterization of STEC isolates [8].

The presence of these pathogens in the food chain, together with the widespread and uncontrolled use of antibiotics, has led to an increase in antibiotic resistance among the members of *Enterobacteriaceae* family [9,10]. Antibiotic resistance, particularly to β-lactam antibiotics, is primarily mediated by enzymes such as extended-spectrum β-lactamases (ESBLs) and plasmid-mediated AmpC β-lactamases (pAmpC), which significantly limit available treatment options [11].

According to the Ambler classification system, β-lactamases are divided into four classes (A–D) based on their molecular structure. Class A includes TEM-, SHV- and CTX-M-type β-lactamases encoded by *bla_TEM_*, *bla_SHV_* and *bla_CTX-M_* genes, respectively. Class D comprises OXA-type β-lactamases, which have the ability to hydrolyze oxacillin and cloxacillin and are not inhibited by clavulanic acid [12]. AmpC β-lactamases belong to class C and are typically chromosomally encoded. However, they may also be carried on the plasmids and be transferred by horizontal transmission between many Gram-negative bacteria. Although previously ESBL- and pAmpC-producing bacteria have been mainly related to hospital associated infections, nowadays they are commonly detected at early stages of food processing [10,12,13]. The dissemination of ESBL-producing *Enterobacteriaceae* is largely facilitated by plasmid-mediated horizontal gene transfer, enabling the spread of resistance determinants among bacterial populations associated with animals, foods, humans, and the environment [14].

Data from global studies indicate that the prevalence of ESBL- and plasmid-mediated AmpC (pAmpC)-producing *Salmonella* spp. and *E. coli*, particularly in poultry but also in bovine meat and pork, is increasing. While ESBL- and AmpC-producing bacteria have been documented in poultry, sheep, and bovine-derived products in Türkiye [10,15,16,17,18,19], comprehensive characterization of antimicrobial resistance in bovine carcasses at the primary production stage specifically, during slaughter operations remains poorly defined. Although ESBL-producing *E. coli* have been reported from sheep (carcass, cecal content, and fecal samples) and slaughterhouse environments in Türkiye [20], comprehensive data on ESBL and pAmpC production in *Salmonella* spp. and *E. coli* O157:H7 isolated from bovine carcasses remain limited. Notably, no studies have comprehensively characterized ESBL and pAmpC β-lactamases in *S*. Enteritidis, *S*. Typhimurium, and *E. coli* O157:H7 isolated from bovine carcasses in Türkiye. Determining the prevalence of antimicrobial-resistant bacteria at the early stages of the food chain is therefore of critical importance for identifying contamination sources and assessing consumer exposure. This study determined the prevalence and characterized the molecular and phenotypic antimicrobial resistance profiles of ESBL- and pAmpC-producing *S*. Enteritidis, *S*. Typhimurium, and *E. coli* O157:H7 isolated from bovine carcasses at slaughter in Türkiye.

## 2. Results

### 2.1. Isolation and Molecular Characterization of Isolates

**Figure 1:**
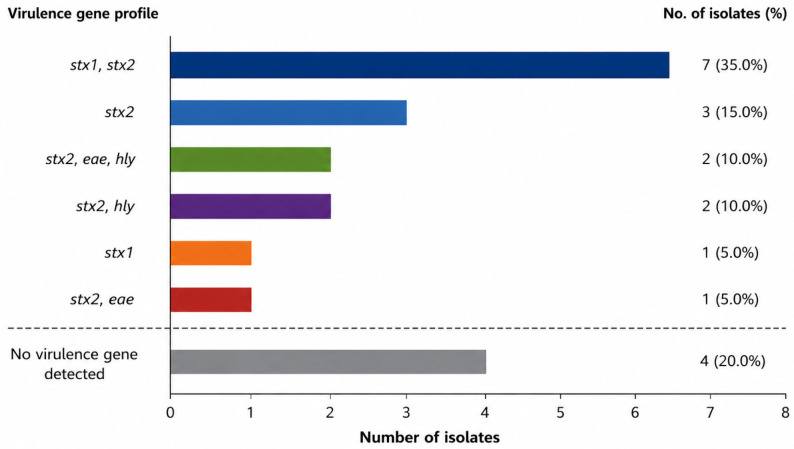
Distribution of virulence gene profiles among *E. coli* O157:H7 isolates (*n* = 20).

A total of 25 *Salmonella* spp. (8.3%) and 20 *E. coli* O157:H7 isolates (6.6%) were confirmed by PCR from 300 bovine carcasses examined. All 25 presumptive *Salmonella* isolates were confirmed by biochemical and serological testing, followed by molecular confirmation using 16S rRNA PCR. The prevalence of *S*. Enteritidis and *S*. Typhimurium was 32% (8 isolates) and 68% (17 isolates), respectively. It was also observed that all of the *E. coli* O157:H7 isolates carried the *eco1*, *eco2* genes, *rfbE* and *fliCh7*. Sixteen out of 20 *E. coli* O157:H7 isolates (80%) were positive for at least one of the virulence genes (*stx1*, *stx2*, *eae*, and *hly*) investigated. The most frequently detected virulence gene was *stx2* (15 isolates) followed by *stx1* (8 isolates), *hly* (4 isolates), and *eae* (3 isolates). The virulence gene combinations are presented at Figure 1.

### 2.2. Phenotypic and Genotypic Characterization of ESBL Production

Phenotypic testing by combination disk diffusion of *Salmonella* spp. and *E. coli* O157:H7 isolates showed that 31 of 45 isolates (68.8%) were ESBL-positive. Among the *Salmonella* isolates (*n* = 25), 16 isolates (64%) were ESBL-positive, including 11 of 17 *S*. Typhimurium isolates (64.7%) and 5 of 8 *S.* Enteritidis isolates (62.5%). Moreover, 14 *E. coli* O157:H7 isolates (70%) were found to be phenotypically ESBL-positive.

In the present study, isolates were evaluated for their phenotypic resistance profiles and ESBL-associated genes (*bla_CTX-M_*, *bla_TEM_*, *bla_SHV_* and *bla_OXA_*). The results revealed that 29 out of 31 phenotypically ESBL-producing isolates also carried at least one of the four ESBL-associated genes investigated. Among 29 ESBL gene-positive isolates, *bla_CTX-M_* was the most frequently detected gene (79.3%), followed by *bla_TEM_* (37.9%), *bla_SHV_* (37.9%), and *bla_OXA_* (3.4%). Multiple ESBL gene combinations were observed in both *Salmonella* spp. and *E. coli* O157:H7 isolates, with a greater variety of combinations detected in *E. coli* O157:H7 isolates. Data on the distribution of the genes investigated are presented in Table 1 and Table 2.

Eighteen out of 23 isolates carrying *bla_CTX-M_* gene were found to harbor at least one of the gene groups (CTX-M1, CTX-M2, CTX-M8, CTX-M9 and CTX-M25) investigated. It was recorded that 17 out of 18 isolates (94.4%) were carrying CTX-M25, 16 isolates (88.8%) CTX-M9, 12 isolates (66.6%) CTX-M1, 11 isolates (61.1%) CTX-M2 and 5 isolates (27.7%) CTX-M8. CTX-M gene group combinations observed in *Salmonella* spp. and *E. coli* O157:H7 isolates are presented in Table 3.

### 2.3. Phenotypic and Genotypic Characterization of pAmpC Production

Thirteen out of 45 isolates (28.8%) showed phenotypic plasmid-mediated AmpC (pAmpC) type β-lactamase production. When it is evaluated by species basis, eight *Salmonella* isolates (32%) (two *S*. Enteritidis and six *S*. Typhimurium), and five *E. coli* O157:H7 isolates (25%) were found to be phenotypically pAmpC-positive. All isolates were further analyzed for the presence of pAmpC-associated gene groups (MOX, CIT, DHA, ACC, EBC, and FOX) Genotypic analysis revealed that 17 of 45 isolates (37.7%) carried at least one pAmpC gene group. When phenotype–genotype compatibility was evaluated, all phenotypically pAmpC-positive *Salmonella* spp. and *E. coli* O157:H7 isolates were also positive at the genotypic level. In addition, four isolates (two from each pathogen) that were phenotypically negative were found to carry pAmpC-associated genes. Overall, 10 *Salmonella* isolates (40%) and 7 *E. coli* O157:H7 isolates (35%) were genotypically positive for pAmpC genes (Table 1 and Table 2). Evaluation of pAmpC gene distribution showed that the FOX gene group was detected in all phenotypically pAmpC-positive isolates. The CIT and DHA gene groups were not detected in any isolate.

### 2.4. Antimicrobial Resistance Profiles

Evaluation of antimicrobial resistance profiles showed that all isolates were found to be resistant to at least 1 antimicrobial agent tested. All of the isolates were susceptible to meropenem, but resistant to pefloxacin. Pefloxacin resistance was followed by gentamicin (93.3%), cefoxitin (55.5%), ceftazidime (42.2%), cefotaxime (40%), chloramphenicol (35.5%), ampicillin (26.6%), cefepime (22.2%), aztreonam (17.7%), and Trimethoprim/sulfamethoxazole (8.8%).

**Figure 2:**
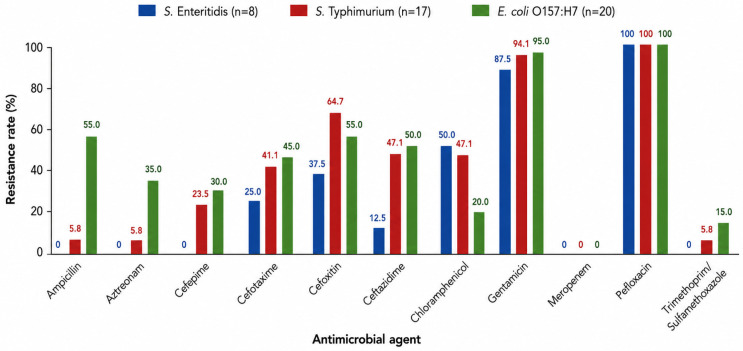
Phenotypic antimicrobial resistance rates (%) of *S.* Enteritidis, *S.* Typhimurium, and *E. coli* O157:H7 isolates against the tested antimicrobial agents.

Species-based evaluation revealed that, in addition to meropenem susceptibility, *S*. Enteritidis isolates were also susceptible to ampicillin, cefepime, aztreonam, and trimethoprim/sulfamethoxazole. *S*. Typhimurium isolates were resistant to at least one of these antimicrobial agents tested. *E. coli* O157:H7 isolates were more resistant to ampicillin (55%), ceftazidime (50%), aztreonam (35%), cefepime (30%) and trimethoprim/sulphametaxazole (15%) compared to *Salmonella* isolates (Figure 2).

Among the 45 isolates examined, 34 (75.6%) exhibited multidrug resistance (MDR), including 18 *Salmonella* spp. (5 *S*. Enteritidis, 13 *S*. Typhimurium) and 16 *E. coli* O157:H7 isolates. Detailed resistance profiles of ESBL- and pAmpC-positive isolates, as well as isolates carrying no resistance genes, are presented in Table 1, Table 2 and Table 4, respectively.

## 3. Discussion

Despite advances in biosecurity and food safety practices, *Salmonella* spp. remain among the most important foodborne bacterial pathogens, with infected animals and cross-contamination during farming, transportation, and slaughtering contributing to their persistence and dissemination in meat products [20]. *S.* Typhimurium and *S.* Enteritidis are the major serovars associated with foodborne salmonellosis worldwide [21]. The prevalence of *Salmonella* spp. on bovine carcasses varies among different regions of the world, with previous studies reporting contamination rates ranging from 3.3% to 18% [22,23,24,25,26], whereas no contamination was detected in some investigations [26,27,28]. In the present study, *Salmonella* spp. were detected in 25 carcasses (8.3%), consistent with previously reported prevalence rates. Of the isolates, 32% were identified as *S.* Enteritidis and 68% as *S.* Typhimurium, consistent with previous reports indicating the predominance of *S.* Typhimurium in bovine carcasses [3,23,24,26,29]. Cross-contamination during dehiding and evisceration, together with inadequate slaughter hygiene practices, may contribute to *Salmonella* contamination of bovine carcasses [30,31].

The prevalence of *E. coli* O157:H7 on bovine carcasses was found to be 6.6% in the present study. Several studies have reported different prevalence rates [32,33,34,35]. These differences may be attributed to variations in sampling and isolation methods, geographic and climatic conditions, and hygienic practices during slaughter. Among the isolates, 80% carried at least one of the investigated virulence genes (*stx1*, *stx2*, *eae*, or *hly*). The predominance of *stx2* observed in the present study is consistent with previous reports from different geographic regions [34,36,37,38]. Stx2-producing strains have been associated with higher virulence than strains producing only Stx1 or both toxins [34]. However, several studies have reported a higher prevalence of *stx1* than *stx2*. Nehoya et al. [39], Berhilevych et al. [40], and Jajarmi et al. [41] identified *stx1* as the predominant Shiga toxin gene among STEC isolates recovered from raw beef, beef and swine carcasses, and caprine sources, respectively. These findings indicate that the distribution of *stx1* and *stx2* varies among STEC populations from different host species and geographical regions, which may reflect differences in the genetic diversity of circulating strains and the mobility of *stx*-encoding prophages. *hly* and *eae* were detected in only 20% and 15% of isolates, respectively. The lower detection of *hly*, a plasmid-borne gene linked to adhesion and tissue damage [42], may reflect plasmid loss during subculturing [34], while the comparatively low *eae* detection may relate to strain-specific carriage of the LEE pathogenicity island, which disseminates via horizontal gene transfer [43]. In the present study, isolates carrying both *stx2* and *eae* were identified, a virulence profile that has been associated with severe clinical outcomes [42].

β-lactamase production is one of the major resistance mechanisms against β-lactam antibiotics. The widespread use of extended-spectrum cephalosporins in veterinary and human medicine has contributed to the emergence and dissemination of ESBL-producing bacteria [44]. Beta-lactam and quinolone antibiotics are commonly used for the treatment of invasive *Salmonella* infections; however, the increasing prevalence and global dissemination of ESBL-producing *Salmonella* strains have compromised the clinical effectiveness of these antimicrobial agents. Although reports of ESBL-producing *Salmonella* spp. remain less frequent than those involving other members of *Enterobacteriaceae* in Türkiye and worldwide, an increasing number of studies have investigated the occurrence of ESBL-producing *Salmonella* strains isolated from foods of animal origin are gradually increasing [45,46,47,48]. In the present study, 29 of 31 phenotypically ESBL-producing isolates carried at least one of the investigated ESBL genes. Two phenotypically ESBL-positive isolates did not harbor any of the investigated ESBL genes. This finding may be explained by the presence of additional ESBL determinants which were not investigated and/or differences in gene expression levels affecting the phenotypic resistance profile in the present study. Similar discrepancies between phenotypic and genotypic ESBL detection have been reported previously in *Enterobacteriaceae* and reflect the complexity of β-lactam resistance mechanisms [49]. Among these isolates, *bla_CTX-M_* was the most frequently detected gene (79.3%), followed by *bla_TEM_* and *bla_SHV_* (37.9%). This *bla_CTX-M_* dominance contrasts with several studies reporting *bla_TEM_* as the predominant ESBL gene in *Salmonella* spp. from poultry and bovine sources [45,46,47]. ESBL gene profiles may vary according to geographical region, sample source, host species, and local antimicrobial usage practices. Among ESBL-positive *E. coli* O157:H7 isolates, all carried *bla_CTX-M_*, followed by *bla_TEM_* (46.1%) and *bla_SHV_* (38.4%), whereas *bla_OXA_* was not detected. Similar findings have been reported in bovine carcass isolates from Czech Republic and Türkiye, where *bla_CTX-M_* was identified as the predominant ESBL gene, frequently occurring alone or in combination with *bla_TEM_* [50,51]. In contrast, Ahmed and Shimamoto [44] reported a lower prevalence of *bla_CTX-M_* and a predominance of *bla_TEM_* among *E. coli* O157 isolates recovered from bovine carcasses. This predominance of *bla_CTX-M_* is consistent with global trends indicating that CTX-M-type enzymes have largely replaced TEM- and SHV-type ESBLs among foodborne *Enterobacteriaceae* [52], and may be related to the extensive use of extended-spectrum cephalosporins and the efficient dissemination of *bla_CTX-M_* genes through conjugative plasmids and other mobile genetic elements [53,54].

The present study demonstrated that 18 of 23 *bla_CTX-M_* positive isolates harbored at least one of the CTX-M-1, -2, -8, -9, and -25 gene groups, CTX-M-25 being the most prevalent gene group (94.4%). This contrasts with previous reports identifying CTX-M-1 as the predominant group among *Salmonella* spp. and *E. coli* from meat samples in Bangladesh, Italy, and Türkiye [10,17,55,56,57]. CTX-M-9 predominance has also been reported in *E. coli* isolates [47,58], whereas Ahmed and Shimamoto [44] detected CTX-M-3 and CTX-M-15 in *E. coli* O157:H7 isolates. Notably, Adıgüzel et al. [59] reported a comparably high prevalence of the CTX-M-8/25 group (95%) among *Salmonella* isolates from poultry meat in Türkiye. Limited reports are available on CTX-M-25-group enzymes, particularly among food-animal isolates. Navon-Venezia et al. [60] demonstrated that CTX-M-25-type genes were plasmid-mediated and associated with a class 1 integron located downstream of an *ISEcp1* element, facilitating dissemination within and between bacterial species. The unexpectedly high prevalence of the CTX-M-25 group observed in the present study may reflect regional dissemination of plasmids or bacterial clones carrying this gene group, together with horizontal transfer mediated by mobile genetic elements and local antimicrobial selection pressure [13,60]. Further molecular epidemiological investigations are needed to clarify the factors and mechanisms underlying the predominance of CTX-M-25 in the study region.

In the present study, FOX was the predominant pAmpC gene group, followed by EBC, ACC, and MOX, whereas CIT and DHA were not detected. However, several studies investigating pAmpC resistance in foods of animal origin reported CIT, particularly CMY-2 and CMY-4 variants, as the predominant gene group [10,61,62]. Variation in the distribution of pAmpC gene groups may be associated with differences in host species, geographical region, production systems, and antimicrobial usage practices. It should also be noted that cefoxitin resistance is widely used as a preliminary screening criterion for AmpC production; however, cefoxitin-based screening alone may not accurately identify all AmpC-producing isolates. Therefore, pAmpC gene groups were investigated in all isolates regardless of their phenotypic screening results. A total of 17 isolates carried at least one pAmpC gene group, whereas only 13 isolates were classified as putative AmpC producers based on antimicrobial susceptibility profiles. This finding highlights the importance of molecular confirmation and indicates that cefoxitin-based screening alone may underestimate the occurrence of pAmpC determinants.

Antimicrobial resistance (AMR) is recognized as one of the major global public health threats, compromising the effectiveness of antimicrobial therapy and contributing to the worldwide dissemination of multidrug-resistant (MDR) bacteria [63]. Antimicrobial susceptibility testing was performed for all isolates, regardless of their ESBL status, to provide a comprehensive overview of resistance profiles among *Salmonella* spp. and *E. coli* O157:H7 isolates recovered from bovine carcasses. Although ESBL production primarily confers resistance to β-lactam antibiotics, resistance to other antimicrobial classes may occur independently and contribute to multidrug resistance [53]. Therefore, evaluation of the antimicrobial resistance profiles of all isolates may provide a broader understanding of potential resistance sources and resistance dissemination patterns.

In the present study, all isolates were resistant to pefloxacin, whereas all remained susceptible to meropenem. High resistance rates were also observed for gentamicin (93.3%) and cefoxitin (55.5%). All *Salmonella* isolates were resistant to at least one antimicrobial tested, with *S.* Typhimurium isolates exhibiting broader resistance profiles than *S.* Enteritidis isolates. Similar resistance patterns, including resistance to aminoglycosides, quinolones, chloramphenicol, sulfonamides, and β-lactam antibiotics, have previously been reported in *Salmonella* isolates recovered from bovine carcasses [24,64]. Similarly, all *E. coli* O157:H7 isolates were resistant to pefloxacin, with high resistance also observed for gentamicin (95%), ampicillin (55%), cefoxitin (55%), and ceftazidime (50%), and lower resistance for trimethoprim/sulfamethoxazole (15%), broadly consistent with previous reports of variable resistance among *E. coli* O157:H7 isolates against these antimicrobial classes [5,65,66]. Differences among studies may be associated with variations in antimicrobial susceptibility testing methodologies, geographical regions, bacterial populations, and antimicrobial usage practices.

Among the antimicrobials tested, pefloxacin and gentamicin exhibited the most notable resistance patterns. The resistance to pefloxacin observed in the present study was comparable to the high rate reported by Orhan et al. [47] (84.6%). According to EUCAST [67], pefloxacin disk diffusion is recommended as a surrogate marker for detecting decreased fluoroquinolone susceptibility in *Salmonella* spp. Therefore, the resistance to pefloxacin observed in the present study indicates widespread reduced susceptibility to fluoroquinolones among the study isolates. Regarding gentamicin, the resistance rates observed in the present study (93.3% in *Salmonella* spp. and 95% in *E. coli* O157:H7) were substantially higher than those reported in the previous studies [16,19,47,65]. Transferable plasmids and other mobile genetic elements have been shown to facilitate the dissemination and persistence of resistance determinants to multiple antimicrobial classes under antimicrobial pressure [13,54]. Collectively, these findings indicate that resistance to critically important antimicrobials is well established among the foodborne isolates investigated, underscoring the importance of integrated surveillance programs combining phenotypic and molecular approaches to better understand and monitor the dissemination of multidrug resistance throughout the food production chain.

This evaluation demonstrated that ESBL production was not limited to resistance against extended-spectrum β-lactam antibiotics but was also associated with multidrug resistance phenotypes. The high cefotaxime and ceftazidime resistance observed among isolates predominantly carrying *bla_CTX-M_* is consistent with the strong hydrolytic activity of CTX-M enzymes against third-generation cephalosporins [53]. In addition, the high quinolone and aminoglycoside resistance rates observed among ESBL-positive isolates may be related to the frequent coexistence of *bla_CTX-M_* genes with non-β-lactam resistance determinants on mobile genetic elements [13,54], contributing to the emergence and dissemination of multidrug-resistant bacterial populations.

When ESBL and pAmpC were evaluated together, heterogeneous β-lactam resistance profiles were observed among the isolates. ESBL-positive isolates generally exhibited characteristic resistance to third-generation cephalosporins, whereas pAmpC-positive isolates displayed more variable phenotypic resistance patterns, consistent with previous reports indicating that the presence of pAmpC genes does not always correlate directly with phenotypic resistance [68]. Several *Salmonella* isolates carrying pAmpC gene groups remained susceptible to ampicillin and other β-lactam antibiotics, highlighting the complexity of genotype–phenotype relationships. Furthermore, *bla_TEM_*, which is commonly associated with ampicillin resistance [69], was not detected in all ampicillin-resistant isolates. This finding suggests that β-lactam resistance may involve multiple resistance mechanisms rather than a single β-lactamase family.

The prevalence and diversity of multidrug-resistant (MDR) bacteria are increasing worldwide, and the transmission of antibiotic resistance through food has become an important public health concern. Food-producing animals may carry antibiotic-resistant bacteria, and contamination during slaughtering and food processing may facilitate the dissemination of resistant pathogens through the food chain. In the present study, a close relationship has been found between MDR profiles of the isolates and ESBL production. Twenty-six (89.6%) ESBL-positive isolates (15 *Salmonella* spp. and 11 *E. coli* O157:H7) were classified as MDR, whereas MDR rate in ESBL negative isolates was 50% (3 *Salmonella* spp. and 5 *E. coli* O157:H7). Similarly, several studies reported that ESBL genes are frequently co-located with resistance determinants for other antimicrobial classes on mobile genetic elements [15,16]. Considering the importance of bovine meat in the human diet, the presence of MDR bacteria on bovine carcasses may represent a potential public health concern. In agreement with previous study [17], all isolates remained susceptible to meropenem. Although carbapenems are not routinely used in veterinary medicine, the absence of carbapenem resistance among the isolates is encouraging from a public health perspective because these agents remain critically important for the treatment of severe infections in human medicine. Nevertheless, the high prevalence of ESBL-producing and multidrug-resistant foodborne bacteria highlights the importance of continuous surveillance to monitor the dissemination of antimicrobial resistance throughout the food chain and to support risk mitigation strategies [14].

In addition to animal-associated factors, contamination of bovine carcasses may also originate from slaughterhouse equipment, processing environments, and personnel during slaughtering procedures. Variations observed in ESBL and/or pAmpC positivity rates among studies may be associated with differences in antibiotic usage practices in both human medicine and food animal production. This study contributes to the limited data available regarding ESBL-producing *E. coli* O157:H7, *S*. Typhimurium, and *S*. Enteritidis isolates recovered from bovine carcasses in Türkiye. Although previous studies on ESBL-producing foodborne pathogens have mainly focused on poultry and swine, the present study provides additional data regarding the occurrence, antimicrobial resistance profiles, and molecular resistance characteristics of ESBL-producing *Salmonella* spp. and *E. coli* O157:H7 isolates associated with bovine carcasses.

## 4. Materials and Methods

### 4.1. Sampling Sites and Sample Collection

This study was carried out in three slaughterhouses, where routine large animal slaughtering procedures were conducted, located in Aydın and Muğla provinces of Türkiye. Between 2018 and 2020, a total of 300 freshly slaughtered cattle carcasses were randomly selected from routine slaughter batches for sampling. Sampling was performed on multiple occasions throughout the study period. Samples were collected post-evisceration and prior to chilling from a 400 cm^2^ area on the round, flank, brisket, and neck of each carcass according to the TSE EN ISO 17604 standard [70]. Sterile templates (10 cm × 10 cm) and sterile sponge swabs (World Bioproduct SRDRY-G, Woodinville, WA, USA) were used for sample collection.

### 4.2. Salmonella spp. and Escherichia coli O157:H7 Isolation

Isolation of *Salmonella* spp. was performed according to ISO 6579-1:2017 [71]. Sponge swab samples were pre-enriched in Buffered Peptone Water (CM0509, Oxoid, Basingstoke, UK) and incubated at 37 °C for 24 h. Subsequently, 1 mL of the pre-enrichment culture was transferred into Mueller–Kauffmann Tetrathionate Novobiocin broth (MKTTn; CM1048, Oxoid, Basingstoke, UK), and 0.1 mL was transferred into Rappaport–Vassiliadis Soya broth (RVS; CM0866, Oxoid, Basingstoke, UK). The inoculated broths were incubated at 37 °C and 41.5 °C for 24 ± 3 h, respectively. Selective enrichment cultures were inoculated onto Xylose Lysine Deoxycholate agar (CM0469, Oxoid, Basingstoke, UK) and Brilliant Green Agar (CM0329, Oxoid, Basingstoke, UK) and incubated at 37 °C for 24 h. Presumptive *Salmonella* colonies were purified on Nutrient Agar (CM0003, Oxoid, Basingstoke, UK) and subjected to biochemical confirmation using Triple Sugar Iron Agar (TSI; CM0277, Oxoid, Basingstoke, UK), Lysine Iron Agar (LIA; CM0381, Oxoid, Basingstoke, UK), and Urea Broth (CM0071, Oxoid, Basingstoke, UK), followed by the Salmonella Latex Test (FT0203A, Oxoid, Basingstoke, UK) and PCR confirmation.

Isolation of *E. coli* O157:H7 was performed as previously described by the FDA BAM:2016 [72]. Sponge swab samples were enriched in modified Tryptone Soya Broth (mTSB; CM0989, Oxoid, Basingstoke, UK) supplemented with 10 mg/L novobiocin (Novobiocin Selective Supplement; SR0181, Oxoid, Basingstoke, UK) and incubated at 37 °C for 24 h. Following enrichment, cultures were inoculated onto Cefixime–Tellurite Sorbitol MacConkey agar prepared from Sorbitol MacConkey agar base (CM0813, Oxoid, Basingstoke, UK) supplemented with Cefixime Tellurite Selective Supplement (SR0172E, Oxoid, Basingstoke, UK) and incubated at 37 ± 1 °C for 18–24 h. Presumptive colonies exhibiting morphology consistent with *E. coli* O157:H7 on CT-SMAC agar were selected and subcultured onto Eosin Methylene Blue agar (EMB; CM0069, Oxoid, Basingstoke, UK) for purification. Purified isolates were subjected to conventional biochemical characterization, including lactose fermentation and IMViC tests, and screened for the O157:H7 antigen using the Remel™ Wellcolex™ *E. coli* O157:H7 latex agglutination test (Remel, Lenexa, KS, USA). Isolates identified as *E. coli* O157:H7 were subsequently confirmed by PCR.

Only one representative isolate was selected from each positive carcass for further characterization, molecular analysis, and antimicrobial susceptibility testing. Confirmed isolates were stored in Brain Heart Infusion broth (BHI; CM1135, Oxoid, Basingstoke, UK) supplemented with 20% (*v*/*v*) glycerol (1.04057.2511, Merck, Darmstadt, Germany) at −20 °C until further analysis.

### 4.3. Molecular Characterization and Confirmation of Isolates

Total DNA extraction of *Salmonella* spp. and *E. coli* O157:H7 isolates obtained from conventional bacteriological methods was conducted by using Qiagen MagAttract HMW DNA Kit (Qiagen, Hilden, Germany) according to the manufacturer’s instructions. The presence of *Salmonella enterica* 16S rRNA gene in *Salmonella* isolates was confirmed according to the PCR protocol applied by Lin and Tsen [73]. Serotype-specific identification of *Salmonella* Enteritidis and *Salmonella* Typhimurium was performed as described by Alvarez et al. [74]. Conventionally described *E. coli* O157:H7 isolates were confirmed by using Parvin et al. [56] method in which the presence of *eco1* and *eco2* genes were determined. Then the presence of the *rfbE* (O157) gene was determined by monoplex PCR [75], whereas the *fliCh7* (H7), *stx1* (Shiga toxin 1), *stx2* (Shiga toxin 2), *eae* (intimin), and *hly* (hemolysin) genes were detected by multiplex PCR [76].

### 4.4. Antimicrobial Resistance Tests

#### 4.4.1. Determination of Phenotypic Antimicrobial Resistance

The antimicrobial resistance profiles of isolates were determined by using Kirby-Bauer disk diffusion method. Tests were conducted according to the criteria suggested by EUCAST [67]. For this purpose, ampicillin (10 μg), cefotaxime (5 µg), cefoxitin (30 µg), ceftazidime (10 µg), cefepime (30 µg), meropenem (10 µg), aztreonam (30 µg), pefloxacin (5 µg), gentamicin (10 µg), chloramphenicol (30 µg), and trimethoprim- sulfamethoxazole (1.25–23.75 µg) antibiotic disks (Liofilchem, Roseto degli Abruzzi, Italy) were used. The results were evaluated according to the EUCAST Clinical Breakpoint Tables, Version 14.0 [67]. Multidrug resistance (MDR) was defined as resistance to at least one agent in three or more different antimicrobial classes [77].

#### 4.4.2. Phenotypic Detection of ESBL and AmpC β-Lactamases

Phenotypic detection of ESBL production was performed using the combination disk test. Cefotaxime (5 µg) and ceftazidime (10 µg) disks were tested alone and in combination with clavulanic acid (10 µg). An increase of ≥5 mm in inhibition zone diameter for the combination disks compared with the corresponding cephalosporin disks was interpreted as ESBL positivity [78].

AmpC β-lactamase production was evaluated based on antimicrobial susceptibility profiles. Isolates resistant to cefoxitin but susceptible to cefepime were considered as putative AmpC producers [11,79].

#### 4.4.3. Molecular Detection of ESBL and pAmpC β-Lactamase Genes

The presence of resistance genes (*bla_SHV_*, *bla_TEM_*, *bla_CTX-M_*, and *bla_OXA_*) associated with ESBL production was investigated using multiplex PCR suggested by Fang et al. [80] with minor modifications. Isolates with *bla_CTX-M_* gene, CTX-M gene groups (CTX-M-1, CTX-M-2, CTX-M-8, CTX-M-9, and CTX-M-25) were determined by using multiplex PCR protocols as described by Woodford et al. [81]. Determination of gene groups (MOX, CIT, DHA, ACC, EBC and FOX) responsible for plasmid-mediated AmpC resistance were detected by using multiplex PCR method described by Perez-Perez and Hanson [82]. PCR products were analyzed by agarose gel electrophoresis.

The primer sequences for monoplex/multiplex PCR are presented at Table 5 Detailed PCR reaction mixtures and amplification conditions for all PCR assays used in this study are provided in Supplementary Table S1.

*Salmonella enterica* ATCC 13076, *Escherichia coli* O157:H7 ATCC 43895, and *Klebsiella pneumoniae* NCTC 13440 were used as positive controls, while sterile distilled water was used as a negative control in all PCR assays.

### 4.5. Statistical Analysis

Statistical analyses were performed using IBM SPSS Statistics for Windows (Version 22.0, IBM Corp., Armonk, NY, USA). The prevalence of *Salmonella* spp. and *E. coli* O157:H7, virulence gene profiles (*stx1*, *stx2*, *eae*, and *hly*), ESBL and pAmpC phenotypes, antimicrobial resistance profiles, and resistance gene distributions were analyzed using descriptive statistics and expressed as frequencies (*n*) and percentages (%).

## 5. Conclusions

The findings of the present study indicate that bovine carcasses are important not only as potential sources of foodborne pathogens, but also as vehicles for the dissemination of antimicrobial-resistant bacteria through the food chain. The detection of ESBL-producing and multidrug-resistant *Salmonella* spp. and *E. coli* O157:H7 isolates highlights the potential public health risks associated with the transmission of resistant pathogens via contaminated meat. These results emphasize the importance of regular and systematic antibiotic resistance monitoring programs, together with integrated surveillance strategies for the early detection and control of ESBL-producing and multidrug-resistant bacteria. Within this context, implementation of the One Health approach, considering the interconnected relationships between animal health, food safety, and human health, remains critically important for limiting the dissemination of antibiotic-resistant pathogens and protecting public health.

## Figures and Tables

**Table 1:** Antimicrobial resistance profiles and extended-spectrum β-lactamase (ESBL)/plasmid-mediated AmpC β-lactamase (pAmpC) gene distribution of *Salmonella* isolates.

Isolate No.	Bacteria	AMR Profile	ESBL Genes	pAmpC Group	MDR
S1	*S*. Typhimurium	CN, FOX, PEF	-	FOX	+
S2	*S*. Typhimurium	CN, FOX, PEF	*bla_SHV_*	FOX, EBC, ACC, MOX	+
S3	*S*. Typhimurium	CN, FEP, FOX, PEF,	*bla_TEM_*		+
S4	*S*. Typhimurium	C, CAZ, CN, FOX, PEF	*bla_CTX-M_*	FOX, ACC	+
S5	*S.* Enteritidis	CN, PEF	*bla_CTX-M_*		
S6	*S*. Typhimurium	ATM, C, CAZ, CN, CTX, FOX, PEF	*bla_SHV_*	ACC, FOX	+
S7	*S.* Enteritidis	C, CN, PEF	*bla_SHV_*, *bla_CTX-M_*	-	+
S8	*S*. Typhimurium	CAZ, CN, CTX, FOX, PEF	*bla_TEM_*	FOX, ACC, EBC	+
S9	*S*. Typhimurium	CAZ, CN, CTX, FOX, PEF	*bla_SHV_*, *bla_CTX-M_*	FOX, ACC, EBC	+
S10	*S*. Typhimurium	C, CAZ, CN, CTX, FEP, FOX, PEF	*bla_SHV_*, *bla_CTX-M_*, *bla_OXA_*	-	+
S11	*S.* Enteritidis	C, CN, FOX, PEF	*bla_CTX-M_*	FOX	+
S13	*S.* Enteritidis	C, CN, PEF	*bla_TEM_*	-	+
S16	*S*. Typhimurium	C, CN, PEF, STX	*bla_CTX-M_*, *bla_TEM_*	-	+
S17	*S*. Typhimurium	C, CN, PEF	*bla_CTX-M_*	-	+
S19	*S*. Typhimurium	AMP, C, CN, PEF	*bla_SHV_*	FOX	+
S20	*S*. Typhimurium	C, CAZ, CN, CTX, FEP, FOX, PEF	*bla_CTX-M_*, *bla_TEM_*	FOX, EBC	+
S21	*S.* Enteritidis	C, CN, CTX, FOX, PEF	*bla_CTX-M_*	FOX, EBC	+

AMP: ampicillin, ATM: aztreonam, C: chloramphenicol, CAZ: ceftazidime, CN: gentamicin, CTX: cefotaxime, FEP: cefepime, FOX: cefoxitin, PEF: pefloxacin, STX: trimethoprim/sulfamethoxazole.

**Table 2:** Antimicrobial resistance profiles and ESBL/pAmpC gene distribution of *E. coli* O157:H7 isolates.

Isolate No.	AMR Profile	ESBL Genes	pAmpC Group	MDR
E23	CN, FOX, PEF	*bla_SHV_*, *bla_CTX-M_*	FOX	+
E26	CAZ, CN, PEF	*bla_SHV_*, *bla_CTX-M_*		+
E30	CN, PEF	-	FOX, ACC	
E31	AMP, ATM, C, CAZ, CN, CTX, FEP, FOX, PEF	*bla_SHV_*, *bla_CTX-M_*, *bla_TEM_*	-	+
E32	AMP, ATM, CAZ, CN, CTX, FEP, FOX, PEF	*bla_CTX-M_*, *bla_TEM_*	-	+
E33	AMP, ATM, C, CAZ, CN, CTX, FEP, FOX, PEF, STX	*bla_CTX-M_*, *bla_TEM_*	-	+
E34	AMP, ATM, CAZ, CN, CTX, FOX, PEF, STX	*bla_CTX-M_*, *bla_TEM_*	FOX, ACC	+
E35	AMP, ATM, FOX, CN, CTX, PEF	*bla_CTX-M_*	FOX, EBC	+
E36	AMP, CAZ, CTX, FEP, FOX, PEF	*bla_SHV_*, *bla_CTX-M_*	FOX, EBC	+
E38	AMP, CN, FOX, PEF	*bla_CTX-M_*	FOX, EBC	+
E39	AMP, CN, PEF	*bla_CTX-M_*, *bla_TEM_*	-	+
E41	CN, PEF	*bla_CTX-M_*	-	
E42	AMP, CAZ, CN, FOX, PEF	*bla_SHV_*, *bla_CTX-M_*, *bla_TEM_*	FOX, EBC	+
E43	CN, PEF	*bla_CTX-M_*	-	

AMP: ampicillin, ATM: aztreonam, C: chloramphenicol, CAZ: ceftazidime, CN: gentamicin, CTX: cefotaxime, FEP: cefepime, FOX: cefoxitin, PEF: pefloxacin, STX: trimethoprim/sulfamethoxazole.

**Table 3:** Distribution of CTX-M gene group combinations among *Salmonella* spp. and *E. coli* O157:H7 isolates.

Resistance Genes Combinations	*Salmonella* spp. (*n* = 9)		*E. coli* O157:H7 (*n* = 9)	Total (*n* = 18)
	*S.* Enteritidis (*n* = 2)	*S.* Typhimurium (*n* = 7)		
CTX-M1 + CTX-M9	0	1	0	1
CTX-M1 + CTX-M25	0	0	1	1
CTX-M2 + CTX-M25	0	1	0	1
CTX-M1 + CTX-M9 + CTX-M25	0	0	3	3
CTX-M2 + CTX-M9 + CTX-M25	2	2	1	5
CTX-M1 + CTX-M2 + CTX-M9 + CTX-M25	0	1	1	2
CTX-M1 + CTX-M8 + CTX-M9 + CTX-M25	0	0	2	2
CTX-M1 + CTX-M2 + CTX-M8 + CTX-M9 + CTX-M25	0	2	1	3

**Table 4:** Antimicrobial resistance profiles of isolates lacking ESBL and pAmpC genes.

Isolate No.	Bacteria	AMR Profile	MDR
S12	*S*. Typhimurium	C, CAZ, CN, CTX, FOX, PEF	+
S14	*S*. Typhimurium	CN, PEF	
S15	*S*. Typhimurium	CN, PEF	
S18	*S.* Enteritidis	CAZ, CN, CTX, FOX, PEF	+
S22	*S*. Typhimurium	CAZ, CN, CTX, FEP, FOX, PEF	+
E24	*S.* Enteritidis	CN, PEF	
E25	*E. coli* O157:H7	CN, PEF	
E27	*E. coli* O157:H7	CN, CTX, PEF	+
E28	*E. coli* O157:H7	CAZ, CN, FOX, PEF	+
E29	*E. coli* O157:H7	AMP, ATM, C, CAZ, CN, CTX, FEP, FOX, PEF	+
E37	*E. coli* O157:H7	C, CN, PEF	+
E40	*E. coli* O157:H7	AMP, ATM, CAZ, CN, CTX, FEP, PEF, STX	+
S44	*S.* Enteritidis	PEF	
S45	*S*. Typhimurium	PEF	

AMP: ampicillin, ATM: aztreonam, C: chloramphenicol, CAZ: ceftazidime, CN: gentamicin, CTX: cefotaxime, FEP: cefepime, FOX: cefoxitin, PEF: pefloxacin, STX: trimethoprim/sulfamethoxazole.

**Table 5:** Targets, primer sequences and product sizes.

Target Gene	Primer Sequence (5′-3′)	Amplicon Size (bp)	Annealing Temperature (°C)	Reference
16S rRNA	TGT TGT GGT TAA TAA CCG CA CAC AAA TCC ATC TCT GGA	574	54	[73]
*S.* Enteritidis	TGT GTT TTA TCT GAT GCA AGA GG TGA ACT ACG TTC GTT CTT CTG G	304	57	[74]
*S.* Typhimurium	TTG TTC ACT TTT TAC CCC TGA A CCC TGA CAG CCG TTA GAT ATT	401		
*eco 1* *eco 2*	GACCTCGGTTTAGTTCACAGA CACACGCTGACGCTGACCA	585	68	[56]
*rfbE*	CAGGTGAAGGTGGAATGGTTGTC TTAGAATTGAGACCATCCAATAAG	296	64	[75]
*fliC_h7_*	GCGCTGTCGAGTTCTATCGAGC CAACGGTGACTTTATCGCCATTCC	625	57	[76]
*stx1*	TGTAACTGGAAAGGTGGAGTATACA GCTATTCTGAGTCAACGAAAAATAAC	210		
*stx2*	GTTTTTCTTCGGTATCCTATTCC GATGCATCTCTGGTCATTGTATTAC	484		
*eae*	ATTACCATCCACACAGACGGT ACAGCGTGGTTGGATCAACCT	397		
*hly*	ACGATGTGGTTTATTCTGGA CTTCACGTCACCATACATAT	166		
*bla_SHV_*	CTT TAT CGG CCC TCA CTC AA AGG TGC TCA TCA TGG GAA AG	237	62	[80]
*bla_TEM_*	CGC CGC ATA CAC TAT TCT CAG AAT GA ACG CTC ACC GGC TCC AGA TTT AT	445		
*bla_CTX-M_*	ATG TGC AGY ACC AGT AAR GTK ATG GC TGG GTR AAR TAR GTS ACC AGA AYC AGC GG	593		
*bla_OXA_*	ACA CAA TAC ATA TCA ACT TCG C AGT GTG TTT AGA ATG GTG ATC	813		
CTX-M Group 1	AAA AAT CAC TGC GCC AGT TC AGC TTA TTC ATC GCC ACG TT	415	55	[81]
CTX-M Group 2	CGA CGC TAC CCC TGC TAT T CCA GCG TCA GAT TTT TCA GG	552		
CTX-M Group 8	TCG CGT TAA GCG GAT GAT GC AAC CCA CGA TGT GGG TAG C	666		
CTX-M Group 9	CAA AGA GAG TGC AAC GGA TG ATT GGA AAG CGT TCA TCA CC	205		
CTX-M Group 25	GCA CGA TGA CAT TCG GG AAC CCA CGA TGT GGG TAG C	327		
MOX	GCTGCTCAAGGAGCACAGGAT CACATTGACATAGGTGTGGTGC	520	59	[82]
CIT	TGGCCAGAACTGACAGGCAAA TTTCTCCTGAACGTGGCTGGC	462		
DHA	AACTTTCACAGGTGTGCTGGGT CCGTACGCATACTGGCTTTGC	405		
ACC	AAC AGC CTC AGC AGC CGG TTA TTCGCCGCAATCATCCCTAGC	346		
EBC	TCGGTAAAGCCGATGTTGCGG CTTCCACTGCGGCTGCCAGTT	302		
FOX	AACATGGGGTATCAGGGAGATG CAAAGCGCGTAACCGGATTGG	190		

## Data Availability

The data presented in this study are available upon request from the corresponding author.

## Supplementary Materials

The following supporting information can be downloaded at: https://www.mdpi.com/article/10.3390/antibiotics15070658/s1, Table S1. PCR reaction mixtures and amplification conditions used in this study.
