# Supplementary material for: ESBL- and pAmpC-Producing Salmonella spp. and Escherichia coli O157:H7 Isolated from Bovine Carcasses in Türkiye
Source: Antibiotics (Basel). 2026 Jul 3;15(7):658. doi: 10.3390/antibiotics15070658 (PMC13406030; doi:10.3390/antibiotics15070658)
Supplement: Supplementary file 1 [file antibiotics-15-00658-s001.zip › antibiotics-4376459-supplementary.pdf]

Supplementary Table S1. PCR reaction mixtures and amplification conditions used in this study.

Target gene(s)	PCR reaction mixture	Cycling conditions	Reference
Salmonella 16S rRNA	10 μL 2× ExPrime Taq Premix, 1 μL each of the forward and reverse primers (40 pmol), 2 μL template DNA, and 6 μL ddH_2O	94°C 2 min; 35 cycles: 94°C 30 s, 54°C 30 s, 72°C 30 s; final extension 72°C 2 min	73
S. Enteritidis and *S.* Typhimurium	10 μL 2× ExPrime Taq Premix, 0.25 μL $MgCl_2$ (50 mM), 1 μL each of the forward and reverse primer mixes (10 pmol), 1 μL template DNA, and 4.75 μL ddH_2O	94°C 2 min; 30 cycles: 94°C 1 min, 57°C 1 min, 72°C 2 min; final extension 72°C 5 min	74
eco1/eco2	10 μL 2× ExPrime Taq Premix, 0.1 μL $MgCl_2$ (50 mM), 1 μL each of the forward and reverse primer (10 pmol), 1 μL template DNA, and 6.9 μL ddH_2O	95°C 5 min; 35 cycles: 94°C 1 min, 68°C 1 min, 72°C 1 min; final extension 72°C 7 min	56
rfbE	10 μL 2× ExPrime Taq Premix, 1 μL each of the forward and reverse primer (10 pmol), 1 μL template DNA, and 7 μL ddH_2O	94°C 3 min; 35 cycles: 94°C 30 s, 64°C 1 min, 72°C 40 s; final extension 72°C 3 min	75
fliCh7, stx1, stx2, eae and *hly*	10 μL 2× ExPrime Taq Premix, 1 μL each of the forward and reverse primer mixes (50 pmol), 3 μL template DNA, and 5 μL ddH_2O	94°C 5 min; 35 cycles: 94°C 30 s, 57°C 1 min, 72°C 1 min; final extension 72°C 10 min	76
bla_{SHV}, bla_{TEM}, $bla_{CTX\text{-}M}$ and bla_{OXA}	12.5 μL 2× ExPrime Taq Premix, 0.5 μL $MgCl_2$ (50 mM), 1 μL each of the forward and reverse primer mixes (10 pmol), 2 μL template DNA, and 3 μL ddH_2O	95°C 3 min; 30 cycles: 94°C 30 s, 62°C 1 min, 72°C 1 min; final extension 72°C 10 min	80
CTX-M groups (Group 1, Group 2, Group 8, Group 9 and Group 25)	12.5 μL 2× ExPrime Taq Premix, 1 μL $MgCl_2$ (50 mM), 1 μL each of the forward and reverse primer mixes (25 pmol), 3 μL template DNA, and 6.5 μL ddH_2O	94°C 5 min; 30 cycles: 94°C 1 min, 55°C 1 min, 72°C 1 min; final extension 72°C 7 min	81
pAmpC genes (MOX, CIT, DHA, ACC, EBC and FOX)	12.5 μL 2× ExPrime Taq Premix, 1 μL $MgCl_2$ (50 mM), 2 μL each of the forward and reverse primer mixes (50 pmol), 3 μL template DNA, and 4.5 μL ddH_2O.	94°C 5 min; 35 cycles: 94°C 1 min, 59°C 1 min, 72°C 1 min; final extension 72°C 7 min	82

All PCR assays were performed using ExPrime Taq Premix (2×) (GeNetBio, Korea). Unless otherwise indicated, PCR reactions were carried out in a final volume of 20 μL. Multiplex PCR assays for CTX-M group determination and pAmpC gene detection were performed in a final reaction volume of 25 μL.
